# Measuring health related quality of life (HRQoL) in Lysosomal Storage Disorders (LSDs): a rapid scoping review of available tools and domains

**DOI:** 10.1186/s13023-024-03256-0

**Published:** 2024-07-04

**Authors:** Emily McDool, Philip Powell, Jill Carlton

**Affiliations:** Sheffield Centre for Health and Related Research (SCHARR), University of Sheffield, Sheffield, England

**Keywords:** Patient reported outcome measures (PROMs), Health-related quality of life (HRQoL), Lysosomal Storage Disorders (LSDs)

## Abstract

**Background:**

Lysosomal storage diseases (LSDs) are a group of rare inherited metabolic disorders, consisting of over 70 diseases that are characterised by lysosomal dysfunction. Due to their varied and progressive symptoms, LSDs have a continual impact on patients’ health-related quality of life (HRQoL). Several recently published studies have provided insight into the HRQoL of individuals with LSDs. However, it is challenging to meaningfully synthesise this evidence, since studies often focus upon a particular type of LSD and / or utilise different self-report questionnaires or patient-reported outcome measures (PROMs) to assess HRQoL.

**Aims:**

The aim of this study was to review the published literature in LSDs, to identify the PROMs which have been used to assess HRQoL and generate a conceptual map of HRQoL domains measured in individuals diagnosed with LSDs.

**Methods:**

Three electronic databases were searched in March 2022. Primary studies of any design which utilised multi-item PROMs to assess at least one aspect of HRQoL in individuals with LSDs since 2017 were identified. Data were extracted to assess both the characteristics of each study and of the PROMs utilised within each study. The extraction of HRQoL domains and synthesis were informed by an a priori framework, inductively modified to reflect data emerging from the identified literature. Selection and extraction was undertaken independently by two reviewers; discrepancies were ratified by a third reviewer.

**Results:**

Sixty nine studies were identified which were published 2017-2022, with a combined total of 52 PROMs (71 variants) used to assess HRQoL in individuals with LSDs. The final extracted HRQoL framework included 7 domains (Activities; Physical sensations; Autonomy; Cognition; Feelings and emotions; Self-identity; Relationships), characterised by 37 sub-domains.

**Conclusions:**

This review highlights the breadth and variety of HRQoL domains assessed in individuals with LSDs, across three broad domains of physical, psychological and social functioning. The resultant framework and mapped PROMs will aid researchers and clinicians in the selection of PROMs to assess aspects of HRQoL in people living with LSDs, based on their conceptual coverage.

**Supplementary Information:**

The online version contains supplementary material available at 10.1186/s13023-024-03256-0.

## Background

Lysosomal storage diseases (LSDs) are a group of rare inherited metabolic disorders, consisting of over 70 diseases that are characterised by lysosomal dysfunction [1, 2]. Most LSDs are progressive in nature and life-limiting, although the rate of progression is variable. The symptoms of LSDs vary depending upon a number of variables, including the age of onset and the type of particular disorder. Symptoms can include seizures, developmental delay, movement disorders, blindness and/or deafness. Other notable clinical characteristics include pulmonary and cardiac problems, enlarged internal organs (such as spleen or liver), and abnormal bone growth [3]. As a consequence of their progressive symptoms, LSDs have a continual impact on patients’ health related quality of life (HRQoL) that may change over time and/or in response to treatments [4].

HRQoL is a broad multidimensional concept that summarises the impact of health and disease on quality of life [5]. It is often considered to consist of three core domains: physical, social, and psychological [6]. It can be difficult to measure, particularly across different rare diseases [7]. HRQoL is often assessed using self-report questionnaires, or patient-reported outcome measures (PROMs) from the patient perspective wherever possible, or otherwise from proxy responders. The impact of LSDs on HRQoL is an emerging field, with a number of recently published studies highlighting the negative impact on individuals with LSDs in areas including, but not limited to, fatigue, pain, mobility, hearing and visual impairments, swallowing, speech, anxiety, independence, emotional wellbeing and daily living activities [8–10]. The impact of LSDs on HRQoL also extends to family and carers [10, 11]. Caring for a patient with LSD impacts HRQoL in areas such as social functioning, emotional/psychological functioning, physical functioning and daily activities [12].

It is challenging to meaningfully synthesise the impact of LSDs on HRQoL since studies often focus upon a particular type of LSD and/or use different PROMs, some of which may only measure selected aspects of HRQoL. Without an adequate idea of the evidence space, it is difficult to gain an accurate interpretation of HRQoL in people living with LSDs and to assess which PROMs are best suited to measuring HRQoL in particular LSDs and potentially across LSDs in general. It is also challenging to identify areas of HRQoL in LSDs considered important for attention by researchers and identify critical gaps and areas for future work on HRQoL in LSDs. This presents a barrier to researchers and clinicians planning work in LSDs, in hindering the optimal selection of PROMs to assess aspects of HRQoL in people living with LSDs, for example as outcome(s) in clinical trials.

The aim of this rapid scoping review was to determine the scope of the evidence on which HRQoL PROMs have been used in LSDs and provide an overview and summary of the domains of the HRQoL which have been assessed. This is an initial step to synthesising a wide body of work (across over 70 diseases) and identifying breadth (including commonalities and discrepancies) in the measurement of HRQoL. Specifically, the objectives of the review were to:

1. Identify which PROMs have been used to assess HRQoL in individuals diagnosed with LSDs;
2. Identify the domains of HRQoL which have been assessed in individuals with LSDs;
3. Generate a conceptual map of HRQoL domains measured in individuals diagnosed with LSDs.

When assessing HRQoL in this review, we consider and operationalise a common definition of HRQoL, which focuses on health-related aspects of quality of life and incorporates physical, psychological and social functioning as the three broad domains [6]. Other aspects which may influence quality of life, but which are not directly related to health, including, but not limited to behaviours, spirituality and beliefs, and finances are not considered as aspects of HRQoL for the purposes of this review.

## Methods

The protocol for this review was registered with the International Prospective Register of Systematic Reviews (PROSPERO) (registration no: CRD42022345989) and can be accessed at: https://www.crd.york.ac.uk/PROSPERO/display_record.php?RecordID=345989.

The manuscript has been written using the PRISMA 2020 reporting guidelines and extension for Scoping Reviews (PRISMA-ScR) Checklist [13, 14]. The review was conducted following best practise guidance in conducting and reporting rapid reviews [15].

### Search strategy

Systematic searches of MEDLINE (via Ovid), Embase (via Ovid) and CINAHL (via EBSCO) were conducted on 21st March 2022 to identify the literature and evidence on HRQoL in LSDs. No restrictions on date or language were applied to the search strategy. The search was developed with an information specialist, in line with best practice [15], and comprised of free-text and thesaurus search terms for: (i) broad and umbrella terms for lysosomal storage diseases; (ii) named terms for over 50 lysosomal storage disorders [1, 2, 16]; and (iii) quality of life search filters, as described and published in Uttley et al [17]. A single search was used, where search terms (i) and (ii) were combined using OR before combining with search term (iii) using AND to identify articles using PROMs to assess HRQoL in individuals with LSDs. The search strategy was peer reviewed by a second information specialist in line with guidance [15] prior to undertaking the full searches. A sample search strategy is provided in Appendix A.

### Study selection

The title and abstracts of records retrieved from the searches were screened for inclusion against the criteria outlined in Table 1. The title and abstract screening process was piloted independently by three reviewers, each assessing the same subsample of 50 randomly selected studies. A subset of the total records (40%) were screened by two reviewers (EM, JC) independently with blinded decision making, following best practice guidance [15], and a third reviewer ratified the inclusion or exclusion of articles where disagreement occurred between the initial two ratings. One reviewer conducted the screening of the remaining title and abstracts, and all excluded abstracts were independently reviewed by a different reviewer to ensure that any potentially relevant articles had not been excluded from the review.

**Table 1:** Inclusion and exclusion criteria of studies

**Inclusion**	**Exclusion**
• Patients: Studies of adults and children of any age with a diagnosis of any LSD • Intervention / exposure: Measures of health related quality of life (HRQoL) • Outcomes: Health related quality of life (HRQoL) • Studies: Quantitative studies published as a full-text original article in English which include study data and use a multi-item PROM to assess HRQoL in people diagnosed with any LSD and produced a quantitative score • Studies published since 2017	• Discussion articles or reviews without study data • Studies published in non-English language • Observational studies of aetiology or onset • Studies which do not assess relevant outcomes or domains of interest i.e. not self or proxy-reported HRQoL • Qualitative studies which do not use quantitative instruments (i.e. questionnaires) to measure HRQoL • Studies published prior to 2017

Full text screening was piloted by three reviewers independently. A subset of 20 articles were selected to represent the range of study designs in the full sample and subsequently screened for eligibility. Discrepancies were reviewed and discussed before the remaining full texts were screened by one reviewer to assess relevance or potential relevance, based upon the characteristics ascertained from the full article and the inclusion and exclusion criteria. Excluded full text articles (i.e., not abstracts) that were in English were independently reviewed by a second reviewer in a similar manner to excluded abstracts to ensure that potentially relevant texts were not excluded from the review and to ensure best practise guidance was adhered to [15].

### Data extraction

Studies selected for inclusion were read in full and study data (i.e. information from the study) was extracted by one reviewer following a pilot of the process. Data was extracted on the study characteristics outlined in Table 2 and Appendix C (e.g. country of study, LSD type studied, sample size), using a data extraction spreadsheet which was developed iteratively and piloted prior to use. Once the data was extracted from all retained texts, a second reviewer independently checked a subset of the extracted data (20%) for accuracy, in line with guidance [15].

**Table 2:** Included studies

**Author & year**	**Study type**	**Country**	**LSD type**	**Total study** **sample size**	**Children** **( <=10 years)**	**Adolescents** **(11-17)**	**Adults** **(18+)**	**Method of HRQoL measurement (e.g. self, proxy)**
Adam et al. 2019 [18]	Cohort study	UK	Alpha-mannosidosis	9	Y	Y	Y	Self & proxy
Alaei et al. 2021 [19]	Clinical trial	Iran	Morquio syndrome (MPS IVA)	10	Y	N	N	Proxy (parent)
Aldenhoven et al. 2017 [20]	Cohort study	Multi-centre - 7 European transplant centres (unspecified)	Hurler syndrome (MPS IH)	63	Y	Y	Y (Max 18)	Proxy (parent)
Ali et al. 2021 [21]	Cross-sectional	USA	Fabry disease	69	N	N	Y	Self
Alioto et al. 2020 [22]	Cross-sectional	USA, Pakistan, Israel, Tunisia, Turkey	Gaucher disease type 1 (GD1) & Fabry disease	32 with GD	Y	Y	Y	Self & Proxy (parent)
Arends et al. 2018 [23]	Cohort study	Multicentre - Netherlands and UK	Fabry disease	286	N	N	Y	Self
Aston et al. 2019 [24]	Tool development	UK	Niemann-Pick disease type C (NPC)	43	Y	Y	Y	Proxy (parent)
Avenali et al. 2019 [25]	Cohort study	UK	Gaucher disease type 1 (GD1)	90 (*N*=90 *N*=31 patients with Gaucher disease type 1 (GD); *N*=29 GBA1 heterozygous carriers (Het GBA group);N=30 controls (HC))	?	?	Y	Self
Barba-Romero et al. 2019 [26]	Cross-sectional	Spain	Fabry disease	33	N	Y (Min 17)	Y	Self
Bitirgen et al. 2021 [27]	Cross-sectional	Unspecified	Fabry disease	28 (*N*=14 Patients, *N*=14 controls)	?	?	Y	Self
Borgwardt et al. 2018 [28]	Cohort study	Denmark	Alpha-mannosidosis	33	Y	Y	Y	Proxy (parent / caregiver)
Bremova-Ertl et al. 2022 [29]	Clinical trial	Germany, Slovakia, Spain, USA & UK	Niemann-Pick disease type C (NPC)	33	Y	Y	Y	Self
Chen et al. 2021 [30]	Case control study	USA and Japan	Morquio syndrome (MPS IVA)	161	Y	Y	Y	Self / Proxy (family)
Chen et al. 2021 [31]	Cross-sectional	China	Pompe disease (late onset) (LOPD)	68	N	N	Y	Self
Cleary et al. 2021 [32]	Cohort study	England	Morquio syndrome (MPS IVA)	55	Y	Y	Y	Self or Proxy (parent / caregiver)
Cohen et al. 2020 [33]	Cohort study	Unspecified	Gaucher disease	48	N	N	Y	Self
de Oliveira Freitas et al. 2017 [34]	Cohort study	Brazil	Gaucher disease	17	N	Y	Y	Self
Demaret et al. 2021 [35]	Cohort study	France	Wolman disease	5	Y	N	N	Self & Proxy (parent)
Devigili et al. 2017 [36]	Cross-sectional	Italy	Gaucher disease type 1 (GD1)	25	N	N	Y	Self
Dinur et al. 2020 [37]	Cross-sectional	Israel	Gaucher disease type 1 (GD1)	192	N	N	Y	Self
Dutra-Clarke et al. 2021 [38]	Cohort study	USA	Fabry disease	26	Y (Min 10)	Y	Y	Self
Elstein et al. 2022 [39]	Tool development	Development - Israel; Content validitiy - USA, France & Israel; Psychometric validation - UK	Gaucher disease type 1 (GD1)	33 (content validation); 46 (psychometric validation)	N	N	Y	Self
Forstenpointner et al. 2019 [40]	Cross-sectional	Unspecified	Fabry disease	183 (Total split into likelihood of Fabry disease *N*=40 likely, *N*=96 possible and *N*=47 unlikely; includes *N*=4 with Fabry (diagnosed))	?	?	Y	Self
Gaisl et al. 2020 [41]	Cohort study	Switzerland	Fabry disease	156 (*N*=52 patients matched with 104 healthy adult controls)	N	N	Y	Self
Ganz et al. 2017 [42]	Cohort study	USA, Canada	Gaucher disease type 1 (GD1)	133	N	N	Y	Self
Haller et al. 2019 [43]	Clinical trial	Participants from USA, Mexico, Brazil or Portugal - completed at USA site	Sly syndrome (MPS VII)	12	Y	Y	Y	Unspecified
Hamed et al. 2019 [44]	Cohort study	USA	Pompe disease (late onset) (LOPD)	29	N	N	Y	Self
Harfouche et al. 2020 [45]	Cross-sectional	USA	Pompe disease (late onset) (LOPD)	30	N	N	Y	Self
Harmatz et al. 2018 [46]	Clinical trial	Unspecified	Alpha-mannosidosis	25 (rhLAMAN-05 study), + 33 (rhLAMAN-10 study)	Y	Y	Y	Unspecified
Holub et al. 2021 [47]	Cross-sectional	Unspecified	Fabry disease	24 (*N*=12 (with FD) matched with *N*=12 healthy controls)	N	N	Y	Self
Hu et al. 2021 [48]	Cross-sectional	China	Gaucher disease; Fabry disease; Pompe disease and Mucopolysaccharidosis (type unspecified)	31 (*N*=5 Gaucher, *N*= 14 Fabry, *N*=4 Pompe, *N*=8 Mucopolysaccharidosis)	Y	Y	Y	Self or proxy (caregiver)
Keidel et al. 2021 [49]	Cross-sectional	Germany	Nephropathic cystinosis (infantile)	43	Y	Y	Y	Self
Korlimarla et al. 2020 [50]	Cohort study	USA and South Africa	Pompe disease (GSD II) - infantile (IPD) and late-onset (LOPD)	21	Y	Y	Y (18 Max)	Proxy (parent / family)
Korver et al. 2020 [51]	Cross-sectional	Netherlands	Fabry disease	81	N	N	Y	Self
Koto et al. 2022 [52]	Cross-sectional	Japan	Fabry disease	8	Y	Y	?	Self
Lehtonen et al. 2018 [53]	Cross-sectional	England	Hurler syndrome (MPS IH)	22	Y	Y	N	Proxy (parent)
Lopez et al. 2020 [54]	Cohort study	Unspecified	Gaucher disease	18	N	Y	Y	Self
Matos et al. 2019 [55]	Cross-sectional	Brazil	Hurler syndrome (MPS IH)	22	Y	Y	Y (21 Max)	Proxy (parent)
Matos et al. 2018 [56]	Cross-sectional	Brazil	Hunter syndrome (MPS II); Maroteaux-Lamy syndrome (MPS VI)	16	Y	Y	Y (21 Max)	Self & Proxy (parent)
Mattera et al. 2018 [57]	Cohort study	UK and USA	Hunter syndrome (MPS II)	51	Y	Y	Y	Self & Proxy (caregiver)
Mobini et al. 2022 [58]	Clinical trial	Iran	Niemann–Pick Disease types A and B	5	Y	Y (12 Max)	N	Proxy (parent)
Nowak et al. 2021 [59]	Cross-sectional	Germany and Switzerland	Fabry disease	124	N	N	Y	Self
Olgac et al. 2021 [60]	Cross-sectional	Turkey	Fabry disease; Gaucher disease; Hurler syndrome (MPS I); Hunter syndrome (MPS II);Maroteaux-Lamy syndrome (MPS VI) ; Pompe disease	32 (*N*=19 patients and *N*=13 parents)	Y	Y	Y	Self & Proxy (parent)
Phillips et al. 2020 [61]	Cohort study	Unspecified	Alpha-mannosidosis	33	Y	Y	Y	Unspecified
Pihlstrom et al. 2021 [62]	Cohort study	Norway	Fabry disease	36	N	N	Y	Self
Pintos-Morell et al. 2018 [63]	Cohort study	Spain	Morquio syndrome (MPS IVA)	7	Y	Y	N	Proxy (parent)
Polistena et al. 2021 [64]	Cross sectional	Italy	Fabry disease	106	Y	Y	Y	Self or Proxy (caregiver)
Politei et al. 2021 [65]	Cohort study	Brazil, Argentina and Colombia	Morquio syndrome (MPS IVA)	18	Y	Y	Y	Unspecified
Qi et al. 2021 [66]	Cross-sectional	China	Gaucher disease type 1,2, 3 and unclear (GD1, GD2, GD3)	89 (*N*=40 (patients), *N*=49 (caregiver))	Y	Y	?	Self & Proxy (caregiver)
Quijada-Fraile et al. 2021 [67]	Cross-sectional	Spain	Morquio syndrome (MPS IVA)	33	N	Y (Min 16)	Y	Self
Remor et al. 2018 [68]	Cross-sectional	Spain	Gaucher disease	20	Y	Y	Y (Max 18)	Self & Proxy (parent)
Riccio et al. 2020 [69]	Cohort study	Italy	Fabry disease	7	N	N	Y	Self
Ripeau et al. 2017 [70]	Cohort study	Argentina and Venezuela	Fabry disease	33	Y (Min 10)	Y	Y	Self
Roca-Espiau et al. 2019 [71]	Case control study	Spain	Gaucher disease type 1 and 3 (GD1; GD3)	47 (*N*=27, control group of *N*=20 healthy matched)	N	N	Y	Self
Rosa 2020 [72]	Cross-sectional	Brazil	Fabry disease	37	?	?	Y	Self
Sadjadi et al. 2020 [73]	Cross-sectional	USA	Nephropathic cystinosis	20	N	N	Y	Self
Scheidegger et al. 2018 [74]	Cohort study	Switzerland	Pompe disease (late onset) (LOPD)	7	?	?	?	Self
Sechi et al. 2020 [75]	Clinical trial	Italy	Pompe disease (late onset) (LOPD)	13	N	N	Y	Self
Sigurdardottir et al. 2021 [76]	Cohort study	Norway	Fabry disease	43	N	N	Y	Self
Suzuki et al. 2020 [77]	Cross-sectional	Japan	Hunter syndrome (MPS II)	109	Y	Y	Y	Self & Proxy (family)
Tantawy et al. 2020 [78]	Cross-sectional	Egypt	Gaucher disease type 1 and 3 (GD1; GD3)	24	N	Y	Y	Self
Vaeggemose et al. 2021 [79]	Case-control study	Denmark and Germany	Pompe disease (late onset (LOPD))	20 (*N*=10 (and *N*=10 matched controls))	N	N	Y	Unspecified
Vallim et al. 2020 [80]	Cross-sectional and case control	Brazil	Fabry disease	16	N	Y (Min 17)	Y	Self
Vallim et al. 2019 [81]	Case-control study	Brazil	Fabry disease	31 (*N*=17 (*N*=17 (11 classic, 6 non-classic) and control group *N*=14)	N	Y (Min 17)	Y	Self
Velicki et al. 2021 [82]	Cohort study	Unspecified	Hurler syndrome (MPS IH); Hurler-Sheie syndrome (MPS IS); Hunter syndrome (MPS II); Mucolipidosis III (ML III)	25 (*N*=6 LSD: *N*=1 Hurler syndrome (MPS IH); *N*=1 Hurler-Sheie syndrome (MPS IS); *N*=3 Hunter syndrome (MPS II); *N*=1 Mucolipidosis III (ML III))	N	Y	Y	Self
Von Cossel 2021 [83]	Cross-sectional	Germany	Fabry disease (non-classical variant)	9	N	N	Y	Self
Wenninger et al. 2019 [84]	Clinical trial	Germany	Pompe disease (late onset) (LOPD)	21	N	N	Y	Self
Wilke et al. 2019 [85]	Cross-sectional	Brazil	Gaucher disease type 1 (GD1)	23	N	N	Y	Self
Yuan et al. 2020 [86]	Cross-sectional	Netherlands	Pompe disease (late onset) (LOPD)	121	N	N	Y	Self

Where it is unclear whether a study includes participants of a specific age range, ‘?’ is entered into the age categories

The study team obtained a copy of each named questionnaire and version which was identified in the full text extraction process as potentially being a PROM which may assess or include items which assess HRQoL. Where necessary, information was sought on the version and use of each instrument by revisiting the studies included in the review and supplementary materials and exploring licensing information or studies outside of the review for further information. Where insufficient information was available on the version or specific PROM utilised, the most likely version was recorded based on the available information (to avoid double-counting of PROMs and domains). Each questionnaire was reviewed by the full study team and consensus was reached on the inclusion eligibility of each PROM. The PROM inclusion criteria is outlined in Appendix B, Table 1. Due to the scope of the review and since a large number of PROMs were used infrequently and lacked recent references, a date restriction was imposed to include papers published since 2017. Therefore, PROMs which were not utilised in the last five years were excluded from the review. The rationale behind this decision related to the relevance of the review to ensure that the focus was on concepts and domains considered relevant within the contemporary HRQoL literature and current research, as older measures may not include concepts which have more recently been considered relevant. Following a review of the PROMs, studies were excluded if they did not include a PROM which met the PROM inclusion criteria (outlined in Appendix B, Table 1).

A separate PROM data extraction spreadsheet was developed iteratively and piloted prior to use. The retained PROMs considered to measure HRQoL were assessed and the characteristics of the PROM were extracted, as outlined in Table 3 and Appendix D. Data was extracted by one reviewer and a subset of data (20%) was independently checked by a second reviewer.

**Table 3:** PROMs included in review

**PROM** **(Questionnaire and version)**	**Abbrev.**	**Freq. studies**	**LSD specific** **PROM**	**PROM focus (Generic HRQoL, condition / illness specific) as described in using studies**	**Designed for use in paediatric population**
Achenbach system of empirically based assessment (ASEBA) Child Behavior Checklist - Age 1.5-5 [53]	CBCL	1	N	Behaviour	Y
Achenbach system of empirically based assessment (ASEBA) Child Behavior Checklist - Age 6-18 [50, 53]	CBCL	2	N	Behaviour	Y
Achenbach system of empirically based assessment (ASEBA) Adult self-report [21]	ASR	1	N	Social-adaptive and psychological functioning	N
Activity of daily living survey [30, 77]	ADL	2	N	ADL	N
Beck Depression Inventory [54, 59, 78, 85]	BDI	4	N	Depression	N
Beck Depression Inventory – ii [25, 32]	BDI-II	2	N	Depression	N
Boston Carpal Tunnel Questionnaire [82]	BCTQ	1	N	Carpal tunnel	N
Brief Pain Inventory Short form [32, 38, 62, 65, 69, 70]	BPI SF	6	N	Pain	N
Brief Pain Inventory [23, 26, 51, 72]	BPI	4	N	Pain	N
Centre for Epidemiological Studies – Depression scale [51]	CES-D	1	N	Depression	N
Childhood Health Assessment Questionnaire [28, 43, 46, 61]	CHAQ	4	N	Generic HRQoL	Y
Composite Autonomic Symptom Scale 31 [27]	COMPASS 31	1	N	Autonomic symptoms	N
Eating Assessment Tool [73]	EAT-10	1	N	Dysphagia	N
Epworth Sleepiness Scale [41, 54, 81, 85]	ESS	4	N	Sleepiness	N
EQ-5D-5L [18, 23, 28, 29, 32, 46, 61, 63]	EQ-5D-5L	7	N	Generic HRQoL	N
EQ-5D-3L [23, 26, 33, 48, 59, 64, 65, 69]	EQ-5D-3L	8	N	Generic HRQoL	N
EQ-5D-Y [18, 29, 48]	EQ-5D-Y	3	N	Generic HRQoL	Y
FabryScan questionnaire [40]	FabryScan	1	Y	Fabry disease	N
Fatigue Severity Scale [54, 74]	FSS	2	N	Fatigue	N
Gaucher Disease type-1-specific Patient Reported Outcome Measure (routine monitoring) [37, 39]	rmGD1-PROM	2	Y	Type 1 Gaucher disease (GD1)	N
Gaucher Disease type-1-specific Patient Reported Outcome Measure (clinical trials) [39]	ctGD1-PROM	1	Y	Type 1 Gaucher disease (GD1)	N
Geriatric Depression Scale [54]	GDS	1	N	Depression	N
Health Assessment Questionnaire [65, 67, 72]	HAQ	3	N	Generic HRQoL	N
Hospital Anxiety and Depression Scale [60, 62]	HADS	2	N	Anxiety & depression	N
HUI3 [18, 57]	HUI3	2	N	Generic HRQoL	N
Kiddo-KINDL-r [52]	Kiddo-KINDL-r	1	N	Generic HRQoL	Y
Kiddy-KINDL-r[52]	Kid-KINDL-r	1	N	Generic HRQoL	Y
Kid-KINDL-r[52]	Kiddy-KINDL-r	1	N	Generic HRQoL	Y
MD. Anderson Dysphagia Inventory [73]	MDADI	1	N	Dysphagia	N
Michigan Hand Outcomes Questionnaire [56]	MHQ	1	N	Hand functioning	N
MPS questionnaire [30]		1	Y	MPS	N
Neuropathic Pain Symptom Inventory [36]	NPSI	1	N	Pain	N
Non Motor symptom Questionnaire [54]	NMSQ	1	N	Non-motor symptoms	N
NPC quality-of-life questionnaires for children [24]	NPCQLQ-C	1	Y	Niemann-Pick type C	Y
NPC quality-of-life questionnaires for adults [24]	NPCQLQ-A	1	Y	Niemann-Pick type C	N
PainDETECT questionnaire of German Research Network on Neuropathic Pain [83]	PD-Q	1	N	Pain	N
Pediatric Quality of Life Inventory 4.0 - Toddler(age 2-4) -parent report [35, 68]	PedsQL 4.0	2	N	Generic HRQoL	Y
Pediatric Quality of Life Inventory 4.0 - Young children (age 5-7) - self [22, 35, 68]	PedsQL 4.0	3	N	Generic HRQoL	Y
Pediatric Quality of Life Inventory 4.0 - Young children (age 5-7) – proxy [22, 35, 68]	PedsQL 4.0	3	N	Generic HRQoL	Y
Pediatric Quality of Life Inventory 4.0 - Child (age 8-12) -self or proxy [22, 35, 55, 68]	PedsQL 4.0	4	N	Generic HRQoL	Y
Pediatric Quality of Life Inventory 4.0 - Teens (age 13-18) - self or proxy [22, 55, 68]	PedsQL 4.0	3	N	Generic HRQoL	Y
Pediatric Quality of Life Inventory 4.0 - Young adults (age 18-25) -self or proxy [22]	PedsQL 4.0	1	N	Generic HRQoL	N
Pediatric Quality of Life Inventory 4.0 - Adults (age 18+) - self or proxy [22]	PedsQL 4.0	1	N	Generic HRQoL	N
Pediatric Quality of Life Inventory Multi-dimensional Fatigue Scale - Toddler(age 2-4) -parent report [43]	Peds QL MFS	1	N	Fatigue	Y
Pediatric Quality of Life Inventory Multi-dimensional Fatigue Scale - Young children (age 5-7) - self [43]	Peds QL MFS	1	N	Fatigue	Y
Pediatric Quality of Life Inventory Multi-dimensional Fatigue Scale - Young children (age 5-7) – proxy [43]	Peds QL MFS	1	N	Fatigue	Y
Pediatric Quality of Life Inventory Multi-dimensional Fatigue Scale - Child (age 8-12) -self or proxy [43]	Peds QL MFS	1	N	Fatigue	Y
Pediatric Quality of Life Inventory Multi-dimensional Fatigue Scale - Teens (age 13-18) - self or proxy [43]	Peds QL MFS	1	N	Fatigue	Y
Pediatric Quality of Life Inventory Multi-dimensional Fatigue Scale - Young adults (age 18-25) -self or proxy [43]	Peds QL MFS	1	N	Fatigue	N
Pediatric Quality of Life Inventory Multi-dimensional Fatigue Scale - Adults (age 18+) - self or proxy [43]	Peds QL MFS	1	N	Fatigue	N
Pediatric Outcomes Data Collection Instrument – adolescent [20]	PODCI	1	N	Pediatric orthopaedics	Y
Pediatric Outcomes Data Collection Instrument – child [20]	PODCI	1	N	Pediatric orthopaedics	Y
Pittsburgh Sleep Quality Index [51, 66, 72, 80]	PSQI	4	N	Sleep quality	N
Pompe Disease Impact Scale [44]	PDIS	1	Y	Pompe disease	N
Pompe Disease Symptom Scale [44]	PDSS	1	Y	Pompe disease	N
PROMIS - Dyspnea Short Form 10a [45]		1	N	Dyspnea	N
PROMIS- Fatigue Short Form 8a [45]		1	N	Fatigue	N
PROMIS - Mobility short form v2.0 [82]		1	N	Mobility	N
PROMIS - Pain Interference Short Form 8a [45, 82]		2	N	Pain	N
PROMIS - Peer relations short form V2.0 [82]		1	N	Relations	N
PROMIS - Physical Function Short Form 20a [45]		1	N	Physical function	N
Quality of vision [49]	QoV, OoVQ	1	N	Vision	N
Rasch-built Pompe-specific Activity Scale [86]	R-PAct	1	Y	Pompe disease - ADL	N
Revised Child Anxiety and Depression scale [60]	RCADS	1	N	Anxiety & depression	Y
Rotterdam Handicap Scale [74, 86]	RHS	2	N	ADL	N
SF-36 - version 1 [34, 38, 39, 41, 42, 47, 51, 62, 65, 66, 69–72, 75, 79, 86]	SF-36	17	N	Generic HRQoL	N
SF-36 - version 2 [76, 86]	SF-36v2	2	N	Generic HRQoL	N
Spielberger State and Trait Anxiety Inventory [54]	STAI	1	N	Anxiety	N
St. George’s Respiratory Questionnaire [84]	SGRQ	1	N	Respiratory & dyspnea	N
TNO-AZL Questionnaire for Preschool Children's Health-Related Quality of Life [19, 58]	TAPQOL	2	N	Generic HRQoL	Y
WHOQOL-BREF [31]	WHOQOL-BREF, WHOQOL-26	1	N	Generic HRQoL	N

### Data analysis

The domains relevant to HRQoL were extracted from each PROM. As a result of the breadth and coverage of domains identified, an a priori framework was used as an initial framework on which to map the HRQoL domains. The framework was developed to identify domains of HRQoL to inform the content of a new generic measure, the EQ-HWB (EQ Health and Wellbeing) [87] which was developed as part of the ‘extending the QALY project’ with the EuroQol group. The higher-level domains were retained and modifications were made to the sub-domains within this framework to ensure it accurately reflected the aspects of HRQoL relevant to individuals with LSDs. The identified sub-domains were mapped and categorised into seven higher-level HRQoL domains including: i) Activities; ii) Physical sensations; iii) Autonomy, iv) Cognition; v) Feelings and emotions; vi) Self-identity; and vii) Relationships. Once the framework was finalised, the framework was applied to the data extraction of all PROMs. The sub-domains from all PROMs were independently extracted and mapped by two reviewers. A third reviewer independently completed extraction where disagreement occurred between the initial extraction. Discussion and group extraction followed within the full study team where agreement was not reached across the reviewers.

## Results

**Fig. 1:**
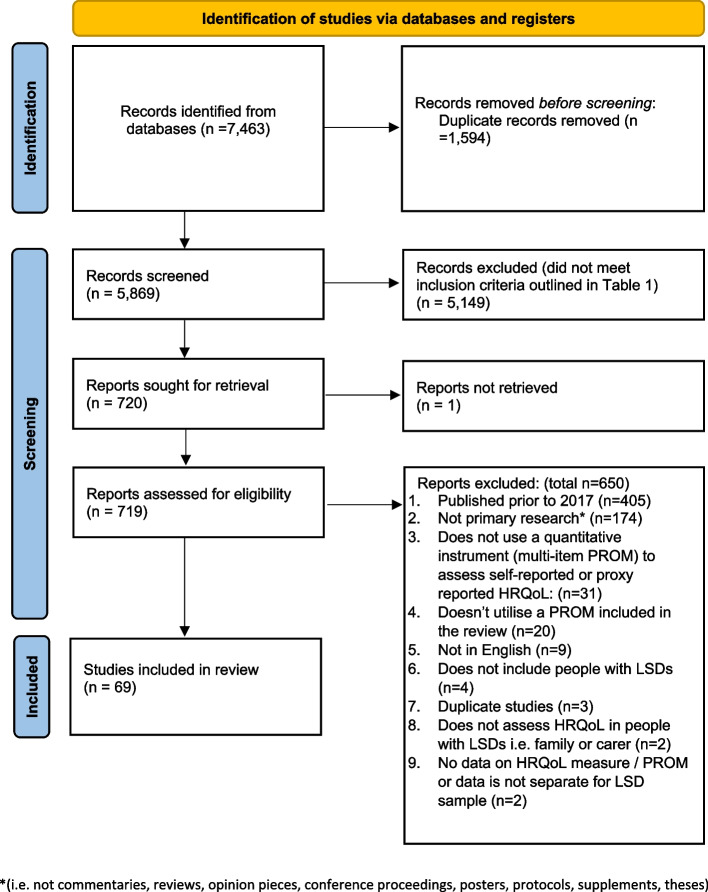
PRISMA flow diagram of studies identified (adapted from [13])

Figure 1 provides an overview of the study selection and screening process. The literature searches initially identified a total of 7,463 records. Removal of duplicate titles resulted in 5,869 records. A further 5,149 records reviewed at the title and abstract stage did not meet the inclusion criteria outlined in Table 1. A total of 69 studies were identified for inclusion within the review, which each utilised one or more of the 52 PROMs (71 variants) retained and assessed in the review.

### Included studies

Table 2 provides the details of the 69 studies included in the review. The majority of studies were described as having a ‘cross-sectional’ design (*n*=30), with the remaining studies described as a ‘cohort study’ (*n*=25), ‘clinical trial’ (*n*=7), ‘case-control’ (*n*=4), ‘tool development’ (*n*=2) or ‘cross-sectional and case control’ study (*n*=1) [80]. There were 30 studies conducted within European countries, 26 in non-European and 5 conducted in or including participants from both European and non-European countries.

The studies focussed on a range of LSDs with 5 studies including multiple LSDs [22, 48, 56, 60, 82]. Broadly categorising the LSD type, the studies included individuals with: Fabry disease (*n*=23), Gaucher disease (*n*=16), Pompe disease (*n*=11), Morquio syndrome (*n*=6), Hurler syndrome & Hurler-Sheie syndrome (*n*=5), Hunter syndrome (*n*=5), Alpha-mannosidosis (*n*=4), Niemann-Pick disease (*n*=3), Nephropathic cystinosis (*n*=2), Maroteaux-Lamy syndrome (*n*=2), Mucopolysaccharidosis (type unspecified) (*n*=1), Mucolipidosis III (*n*=1), Sly syndrome (*n*=1), Wolman disease (*n*=1) (see Table 2 for a more detailed description of the LSD assessed in each paper). The total sample size ranged from 5 to 286 (mean=49 and median=32).

Across the studies, the sample of individuals with LSDs included children (age <11) (*n*=31), adolescents (age 11-17) (*n*=37) and adults (age 18+) (*n*=61). A number of studies included all age groups (*n*=24) and those including children largely also included adolescents (*n*=29/31) and adults (*n*=24/31). Within studies including adults, 5 studies focussed on young adults with inclusion criteria which specified a maximum age of 18 (*n*=3) or 21 (*n*=2) years.

Disease severity was extracted where possible; this naturally varied by study and was measured through various means including the Mainz Severity Score index and disease severity scoring system (DS3) (see Appendix C for details on individual studies).

### Included PROMs

Table 3 provides detail on the 52 PROMs (71 variants) used to measure HRQoL within individuals with LSDs. A range of PROMs were used across the included studies. An average of two PROMs were used per study, while some studies used a single PROM (*n*=30). The version of the PROM used was not specified in some studies (*n*=13) (e.g. some specified the EQ-5D, not the EQ-5D-3L or EQ-5D-5L). The PROMs used in each individual study are outlined in Appendix C.

As detailed in Table 3, the most commonly employed PROM utilised was the SF-36 (version 1), which was used in 17 studies and in four LSDs (Fabry disease, Gaucher disease, Morquio syndrome and Pompe disease (see Appendix B Table 2 which details the PROMs used to measure HRQoL in each LSD group)). Other generic PROMs (*n*=12, *n*=20 variants) were utilised including the EQ-5D-5L and EQ-5D-3L which were used in *n*=7 studies, and *n*=8 studies respectively and across multiple LSD groups.

Nine PROMs were identified which were developed to measure aspects of HRQoL in specific LSDs, including Pompe disease (*n*=3 PROMs), Gaucher disease (*n*=1 PROM, *n*=2 variants) Niemann‑Pick type C (*n*=1 PROM, *n*=2 variants), Fabry disease (*n*=1) and MPS (*n*=1).

A range of specific measures were also used which focussed on a particular condition or aspect of HRQoL. Five PROMs (*n*=11 variants) were described in studies as measures used to assess fatigue, sleepiness or sleep quality. The Pittsburgh Sleep Quality Index (PSQI) and Epworth Sleepiness Scale (ESS) were the most frequently utilised PROMs within this group of PROMs (to assess sleep quality and sleepiness respectively). The Pediatric Quality of Life Inventory Multi-dimensional Fatigue Scale (PedsQL MFS) was used to assess fatigue in a paediatric LSD population (with *n*=5/7 questionnaires for children/adolescents). Studies also assessed mental health in people with LSDs, with PROMs employed to measure depression (*n*=4 PROMs), anxiety (*n*=1 PROM) and both anxiety and depression (*n*=2 PROMs). One anxiety and/or depression PROM was developed for use in paediatric samples (Revised Child Anxiety and Depression scale (RCADS)) whilst the Geriatric Depression Scale (GDS) is generally suited to use in older adults. Although the RCADS was only used in one study, it was administered to the broadest range of LSD types including Fabry disease, Gaucher disease, Hurler syndrome, Hunter syndrome, Maroteaux-Lamy syndrome and Pompe disease. Five PROMs were described as instruments developed to measure pain; the BPI SF was the most commonly used (*n*=6 studies, although with some uncertainty around the version of the BPI). The PROMIS pain interference questionnaire was used in 3 studies to measure pain in broader range of three LSD groups including Hunter syndrome, Hurler syndrome & Hurler-Sheie syndrome and Mucolipidosis III. Other specific areas of health which were the focus of other PROMs included in the review were Dysphagia (*n*=2 PROMs) and Dyspnea / respiratory (*n*=2 PROMs). Some PROMs were used to assess very specific aspects of HRQoL such as the impact of Carpal tunnel (*n*=1 PROMs) and Hand functioning (*n*=1 PROMs) across a range of LSDs (see Table 3).

The number of tems in the PROMs ranged from 5 (EQ-5D-5L, EQ-5D-3L, EQ-5D-Y) to 126 (ASR) (further characteristics of the PROMs included in the review can be found in Appendix D). In some studies, due to multiple PROMs being utilised, as many as 138 items were completed by participants [54]. Where the recall period was specified, the period ranged from current or today to the last 6 months and the response options varied by questionnaire or by question within a questionnaire (e.g., frequency severity). Information on the report type of each PROM was obtained or inferred from the studies in the review; where unavailable, alternative sources were used to extract the information and therefore this may therefore not fully reflect the report type specified at PROM development (e.g. other studies which utilised the instruments, instrument development studies and / or online information from license holders). Questionnaires were designed to be self-report (*n*=38), proxy (*n*=15) or had the option for self and/or proxy report (*n*=18). LSD-specific PROMs were a combination of self-report (*n*=4), proxy report (*n*=2) and self and or proxy (*n*=3).

### HRQoL domains

The HRQoL domains and sub-domains identified across the included studies are provided in Table 4. Thirty-seven sub-domains were identified, mapped, and categorised into 7 higher-order domains of HRQoL including: i) Activities; ii) Physical sensations; iii) Autonomy, iv) Cognition; v) Feelings and emotions; vi) Self-identity; and vii) Relationships. Eight sub-domains were added to reflect the aspects of HRQoL covered by PROMS used in people with LSDs, which were not adequately covered in the original EQ-HWB framework [88], these included: 1) activities of daily living (activities domain); 2) sexual functioning (activities domain); 3) breathing (physical sensations domain); 4) eating, appetite; dribbling and swallowing (physical sensations domain); 5) symptoms (physical sensations domain); 6) understanding (cognition domain); 7) wellbeing and life satisfaction (feelings and emotions domain); and 8) psychological fatigue and energy (feelings and emotions domain). Additional aspects which were not covered in the original framework were combined with existing sub-domains, including fear which was added to the worry (anxiety)/calm sub-domain, and self-confidence which was added to the self-worth/self-respect sub-domain. Two sub-domains were combined following discussion amongst the research team; pain was combined with discomfort since in many cases, it was challenging to distinguish between whether pain or discomfort was assessed (e.g. bloating, joint stiffness, tingling and burning) and discomfort could be interpreted as a continuum of pain. In other commonly utilised HRQoL instruments, pain and discomfort are combined (e.g. EQ-5D-3L, EQ-5D-5L). Thinking clearly and decision making was combined with confusion since overlap and commonalities were identified in these sub-domains. The stigma sub-domain which was included in the original EQ-HWB framework, was not included, as this aspect was interpreted as other people’s perceptions as opposed to the individuals’ own. As a result, embarrassment and self-consciousness and self-esteem were added to the description of the sub-domain self-worth, self-respect and self-confidence to ensure adequate coverage of the individual’s own views.

**Table 4:** HRQoL domains and sub-domains

**Domain**	**Sub-domain**	**Description**
**Activities**	1. Activities of daily living	Day-to-day activities (e.g. feeding, opening items, gripping objects, writing, problems with toileting).
	2. Communication / speech	Speech and verbal communication problems.
	3. Enjoyable or meaningful activities / roles	Sports participation, ability to participate in or impact of health on: activities, school, school work or work.
	4. Hearing	Hearing problems or difficulties.
	5. Mobility	Climbing stairs, walking, running, getting in/out of bed, endurance (walking a distance or at speed), lifting, joint fluidity and range, using mobility devices, standing from sitting.
	6. Self-care	Getting dressed, personal hygiene.
	7. Sexual functioning	Sexual function / interest in sex & satisfaction in sex life.
	8. Vision	Visual problems or difficulties.
**Physical sensations**	1. Breathing	Breathing difficulties, wheezing, breathing aids used, breathlessness.
	2. Eating, appetite, dribbling and swallowing	Eating problems, appetite, dribbling, swallowing.
	3. Fatigue, tiredness and physical weakness	Physical fatigue, lack of energy, tiredness, feeling weak and muscle weakness, exhaustion, worn out.
	4. Pain & discomfort	Pain, discomfort, bloating, dizziness & light headedness, skin problems, joint stiffness swelling, tingling / burning sensation.
	5. Sleep / sleep problems	Sleep / sleep issues.
	6. Symptoms	Digestive problems, changes in appearance (e.g. weight loss or gain and skin changes), sweating, vomiting / nausea.
**Autonomy**	1. Control / choice	Control / lack of control.
	2. Coping	Ability to cope.
	3. Autonomy / independence	Autonomy, independence.
**Cognition**	1. Concentration	Concentration and in/attention.
	2. Memory	Memory / forgetfulness.
	3. Thinking clearly & decision making	Thinking clearly, decision making, confusion.
	4. Understanding	Understanding situations and conversations.
**Feelings & emotions**	1. Anger, frustration & irritability	Anger, tempter, irritability, restlessness, agitation, frustration (e.g. with situation or symptoms), violence.
	2. Guilt / shame	Feelings of guilt or shame.
	3. Hopeless / hope	Pessimism and optimism e.g. about future, hope / hopeless.
	4. Psychological fatigue & energy	Lack of energy or motivation, psychological fatigue.
	5. Sadness (depressed) / happiness	Episodes of crying, self-harm and suicidal thoughts, depression, happiness and sadness.
	6. Vulnerable / safe	Feeling un/safe, vulnerable.
	7. Wellbeing & life satisfaction	Wellbeing positive concepts e.g. enjoyment of life, life satisfaction, enthusiasm, meaningful life.
	8. Worry, anxiety fear & calm	Worry, scared and fearful, anxiety, nervousness, panic, separation anxiety, calm.
**Self-identity**	1. Treated with dignity / respect	Perceptions - treated with dignity / respect.
	2. Self-worth, self-respect and self-confidence	Feeling accomplishment or failure, self-confidence, self-loathing or hatred, worth/ worthlessness, self-esteem, body image / comfort with body, self-consciousness, embarrassment.
**Relationships**	1. Belonging & connectedness	Feeling understood / accepted, feeling included / excluded.
	2. Burden	Burden to others.
	3. Loneliness	Loneliness.
	4. Relationships & friendship	Makes/ has or unable to make friends, impact of health (positive or negative) on relationships / socialising (inc. friends, family), dis/satisfaction with relationships.
	5. Social engagement	Interest in other people, interest in participation or socialising.
	6. Support	Emotional help and support / bonds.

**Fig. 2:**
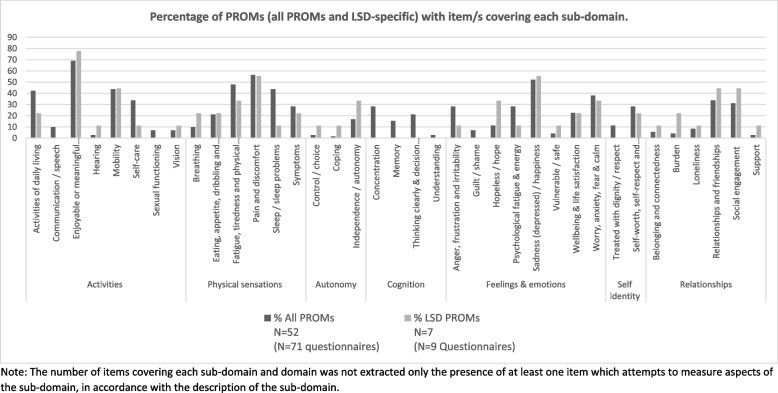
HRQoL domains by PROM group

Figure 2 highlights the frequency or commonality of sub-domains across 1) all PROMs included in the review and 2) LSD-specific PROMs, to compare LSD-specific PROMS to all other PROMs in terms of the aspects of HRQoL they measure. The enjoyable or meaningful activities or roles is the most commonly measured sub-domain with 69% of all PROMs and 78% of LSD-specific PROMs assessing this dimension of HRQoL. Other common sub-domains include pain and discomfort (covered by 56% of PROMs and LSD-specific PROMs) and sadness (depression) and happiness, covered by 52% and 56% of all PROMs and LSD PROMs respectively. Fatigue, tiredness and physical weakness was also commonly assessed in people with LSD (48% of all PROMs and 33% of LSD-specific PROMs), in addition to sleep / sleep problems which was assessed by items in 44% of all PROMs and 11% of LSD-specific PROMs. A high proportion of LSD-specific PROMs also included questions surrounding relationships and friendships (34% of all PROMs and 44% LSD-specific PROMs) and social engagement (31% of all PROMs and 44% LSD-specific PROMs) and these questions were included in the Gaucher Disease type-1-specific Patient Reported Outcome Measure (GD1-PROM) and the NPC quality-of-life questionnaire (NPCQLQ) variants.

The sub-domains that are covered by the PROMs which are used to assess HRQoL in each LSD population (broad categories) are summarised in Table 5. The number of sub-domains covered within each LSD group may be correlated with the types of PROMs utilised, for example generic or specific PROMs, and the number of items within each utilised PROM. However, as is evident in Table 5, some domains are more frequently assessed across different LSD types than others. Activities of daily living, enjoyable or meaningful activities / roles, self-care (activities domain), and pain and discomfort (physical sensations domain) are assessed in all 14 LSD groups. Other sub-domains which are commonly identified across the majority of LSD groups included mobility (activities domain), sleep / sleep problems (physical sensations domain), sadness (depressed) / happiness and worry, anxiety, fear and calm (feelings and emotions domain).

**Table 5:** Domains by LSD

	**Alpha-mannosidosis**	**Fabry disease**	**Gaucher disease**	**Hunter syndrome**	**Hurler syndrome & Hurler-Sheie syndrome**	**Mucolipidosis III**	**Maroteaux-Lamy syndrome**	**Morquio syndrome**	**Mucopolysaccharidosis (unspecified)**	**Nephropathic cystinosis**	**Niemann-Pick disease**	**Pompe disease**	**Sly syndrome**	**Wolman disease**	**Total LSDs (N)**
**Activities**															
Activities of daily living	Y	Y	Y	Y	Y	Y	Y	Y	Y	Y	Y	Y	Y	Y	14
Communication / speech	Y	Y		Y	Y			Y			Y	Y			7
Enjoyable or meaningful activities / roles	Y	Y	Y	Y	Y	Y	Y	Y	Y	Y	Y	Y	Y	Y	14
Hearing	Y			Y				Y							3
Mobility	Y	Y	Y	Y	Y	Y		Y	Y	Y	Y	Y	Y	Y	13
Self-care	Y	Y	Y	Y	Y	Y	Y	Y	Y	Y	Y	Y	Y	Y	14
Sexual functioning		Y	Y		Y			Y				Y			5
Vision	Y	Y	Y	Y				Y		Y					6
**Physical sensations**															
Breathing		Y	Y	Y	Y		Y	Y			Y	Y			8
Eating, appetite, dribbling and swallowing		Y	Y	Y	Y		Y	Y		Y	Y	Y			9
Fatigue, tiredness and physical weakness		Y	Y	Y	Y	Y	Y	Y			Y	Y	Y	Y	11
Pain and discomfort	Y	Y	Y	Y	Y	Y	Y	Y	Y	Y	Y	Y	Y	Y	14
Sleep / sleep problems		Y	Y	Y	Y	Y	Y	Y		Y	Y	Y	Y	Y	12
Symptoms		Y	Y	Y	Y	Y	Y	Y		Y	Y	Y			10
**Autonomy**															
Control / choice											Y	Y			2
Coping											Y				1
Independence / autonomy		Y	Y		Y						Y	Y		Y	6
**Cognition**															
Concentration		Y	Y		Y			Y				Y	Y	Y	7
Memory	Y	Y	Y	Y									Y		5
Thinking clearly & decision making	Y	Y	Y	Y	Y		Y	Y				Y	Y		9
Understanding				Y				Y			Y				3
**Feelings & emotions**															
Anger, frustration and irritability	Y	Y	Y	Y	Y		Y	Y			Y	Y		Y	10
Guilt / shame		Y	Y		Y			Y				Y			5
Hopeless / hope		Y	Y	Y	Y		Y	Y			Y	Y			8
Psychological fatigue & energy		Y	Y	Y	Y		Y	Y			Y	Y	Y		9
Sadness (depressed) / happiness	Y	Y	Y	Y	Y		Y	Y	Y	Y	Y	Y		Y	12
Vulnerable / safe		Y	Y	Y	Y		Y				Y	Y			7
Wellbeing & life satisfaction		Y	Y	Y	Y	Y	Y	Y			Y	Y			9
Worry, anxiety, fear & calm	Y	Y	Y	Y	Y		Y	Y	Y	Y	Y	Y		Y	12
**Self-identity**															
Treated with dignity / respect		Y	Y		Y									Y	4
Self-worth, self-respect and self-confidence		Y	Y	Y	Y		Y	Y		Y	Y	Y			9
**Relationships**															
Belonging and connectedness		Y		Y	Y	Y				Y	Y				6
Burden			Y									Y			2
Loneliness		Y			Y						Y	Y			4
Relationships and friendships		Y	Y	Y	Y	Y		Y		Y	Y	Y		Y	10
Social engagement		Y	Y	Y	Y	Y	Y	Y		Y	Y	Y			10
Support				Y	Y	Y					Y	Y			5
**TOTAL domains per LSD**	13	31	29	28	30	13	19	27	7	15	28	30	11	14	
**% domains covered in LSD**	35%	84%	78%	76%	81%	35%	51%	73%	19%	41%	76%	81%	30%	38%	

The autonomy domain (as a whole) was assessed in the fewest LSD groups (*n*=6 LSD groups) and in the majority of these (*n*=4 LSD groups), only the independence / autonomy sub-domain was evaluated. This is perhaps since autonomy is assessed by a small number of PROMs (*n*=6 PROMs, *n*=14 variants). In Niemann-Pick disease only, all three sub-domains of autonomy were covered. Although LSD-specific PROMs did not assess cognition, this aspect of HRQoL is evaluated across a range of LSD groups (*n*=10 LSDs), using non-LSD-specific PROMs, with thinking clearly and decision making being the most commonly included sub-domain.

The sub-domains assessed by each study in the review, based on the HRQoL themes extracted from the PROMs utilised, are also evaluated and summarised in Appendix E, since some studies use multiple PROMs to assess aspects of HRQoL. While some studies use PROMs to assess a broad range of sub-domains, covering more than 20, other studies focus on a particular element of HRQoL e.g. pain [36] or sleep [80]. There are no studies which utilise PROMs to assess all aspects of HRQoL within the defined framework.

## Discussion

The aim of this rapid scoping review was to identify which PROMs and domains of HRQoL have been assessed in individuals diagnosed with LSDs, and to generate a conceptual framework of HRQoL domains measured in this rare disease area. This framework was designed to be of use to researchers and clinicians in identifying available PROMs for assessing HRQoL domains of interest when working in LSDs. Across the 69 studies included in the review, a range of PROMs (n=52; n=71 variants) were used to assess HRQoL. A conceptual framework of HRQoL was developed including seven domains: i) Activities; ii) Physical sensations; iii) Autonomy; iv) Cognition; v) Feelings and emotions; vi) Self-identity; and vii) Relationships. Within these broad domains, a range of sub-domains were identified spanning a range of different elements of HRQoL and highlighting the complexity and breadth of HRQoL issues associated with LSDs and the different types of LSD.

Within the studies included in the review, a vast range of instruments including generic HRQoL PROMs (e.g. SF-36, EQ-5D-3L, EQ-5D-5L), some of which measure specific aspects of HRQoL (e.g. Brief Pain Inventory; Beck Depression Inventory), and LSD-specific instruments (e.g. Pompe Disease Impact Scale, Niemann-Pick disease type C Quality of Life Questionnaire) were used to assess HRQoL in individuals with LSDs. Additionally, across different LSD groups, a range of instruments were adopted, thus assessing different aspects of HRQoL. This is perhaps unsurprising since LSDs are a group of disorders consisting of over 70 diseases with varying symptoms depending on onset or the particular type of disorder. Therefore, PROMs selected to assess HRQoL may vary depending on the aspects which are relevant to each LSD or appropriate for the population of interest or the intervention question. The PROMs identified included a range of instruments developed for use in adult and paediatric populations. In addition to the diverse symptoms across the different LSDs groups, there is also variance in age of onset and in life expectancy (and life expectancy may depend on the age of onset [89]). Therefore, while a range of PROMs may be used over the life course for some individuals and LSD groups, only PROMs developed for use in paediatric populations may be relevant for some diseases. Across paediatric and adult PROMs, different HRQoL domains are assessed, for example burden is assessed in adults only. Additionally, a high proportion of paediatric measures require, or include, the option for proxy report which can introduce an element of difficulty to assessing some areas of HRQoL as evidence suggests there may be differences between self and proxy reporting with some HRQoL domains being more likely to involve disagreement between self and proxy ratings (e.g. observable vs non-observable domains) [90–92]. As a result of the range of instruments used to measure HRQoL both across and within the different LSD disease groups, it is challenging to synthesise the studies and to summarise the impact of LSDs on HRQoL.

The review thus identified a breadth of HRQoL issues associated with the LSDs, with the most commonly extracted sub-domains including enjoyable or meaningful activities, fatigue, tiredness and physical weakness, pain and discomfort and sadness, depression and happiness. Of the PROMs identified in the review, no single PROM assessed all aspects of HRQoL identified within the framework. Further, generic HRQoL instruments which are frequently adopted within these studies, do not adequately assess all domains and sub-domains of HRQoL. Although some studies utilise multiple PROMs to assess different aspects of HRQoL, no single study assessed all sub-domains of HRQoL, though studies such as clinical trials, for example, may focus on one aspect of health improvement such as pain, rather than an array of outcomes. We may reasonably expect that PROMs are selected to assess the aspects of HRQoL, which are expected to be impacted by LSDs or a specific LSD, or are commonly impacted by LSDs and/or interventions. However, within studies, a sound rationale for the selection of PROMs is required in order to specify the particular HRQoL domains of interest and the appropriateness of the selected PROM in the LSD population. Furthermore, while PROM selection may be determined by the aspects of HRQoL of interest, and a broad selection of PROMs may be necessary, other considerations such as respondent burden may need to be made, especially where multiple PROMs are utilised and/ or PROMs are particularly lengthy but potentially assess the same aspects of HRQoL as viable alternatives. As many as six different PROMs were used in a single study identified within the review, collectively including over 130 items. The effects of respondent burden (e.g. time requirement, anxiety caused and fatigue) may lead to low compliance and data quality [93], and therefore careful considerations need to be made in PROM selection.

In addition to providing rationale for the selection of PROMs, we noted that the reporting of PROMs and versions utilised within some studies was inadequate, for example in some studies it was unclear whether the EQ-5D-3L or EQ-5D-5L was used. Additional reporting detail is required on the version/s of the PROMs due to the differences across questionnaires. As a consequence of inadequate reporting and a lack of information, some PROMs were excluded from this review, thus highlighting the need for accuracy and transparency in reporting in research.

This rapid scoping review has taken an initial step towards synthesising the substantial body of evidence on the impact of PROMs a broad range of LSDs on HRQoL through identifying the range of PROMs used. The results of this review and initial HRQoL framework are thus a resource for researchers, clinicians, and other stakeholders looking at assessing HRQoL, and provides a basis on which to assess additional PROMs and studies and/or built upon the framework used in identifying domains of HRQoL in this rare disease space in future work. The HRQoL domains identified reflect those frequently assessed by utilised PROMs, however the domains extracted from existing instruments may not necessarily reflect the domains which are most important to people with LSDs or more severely impacted by LSDs. To further develop the conceptual framework of HRQoL in LSDs, it could be useful to engage with patients and stakeholders to determine the framework’s relevance, comprehensiveness, and comprehensibility. This would ensure that it includes domains and sub-domains that are relevant to individuals with LSDs (i.e., relevance), it includes all aspects and domains of HRQoL which are important to individuals with LSDs (comprehensiveness) and describes the domains and sub-domains of HRQoL clearly (comprehensibility). As highlighted previously, this may vary by population, for example with the HRQoL domains for paediatric populations varying from adults, which may be driven by the life expectancy in specific LSD groups. Additionally, there may be some domains which are difficult to report given the reliance upon proxy reporting for paediatric populations and/or those unable to self-complete.

Patient and stakeholder involvement may also be a valuable step in identifying the most appropriate PROMs for use in assessing HRQoL in LSDs or in certain LSD groups, through an evaluation of the acceptability of PROMs. Relatedly, while this review highlights the PROMs frequently used to assess HRQoL within people with LSD, it was beyond the scope of the research to ascertain suitability of use of such PROMs in terms of their reliability and validity and reliability. The use of a PROM may not necessarily imply that there is evidence to support its use. Valuable future work may include a formal assessment of the instruments identified in the current review to determine their suitability to measure HRQoL (or a component thereof) for people with LSDs. We would advocate using COSMIN (COnsensus-based Standards for the selection of health status Measurement INstruments) methodology, which is a structured way of assessing psychometric performance, including content validity. Furthermore, there is scope to evaluate the individual items of content of the identified PROMs in the review using a standardized linking approach using the international Classification of Functioning, Disability and Health (ICF) framework. This may provide deeper insight into the extent to which domains are covered within and across individual PROMs through an assessment of the individual items as opposed to evaluation of the HRQoL domain coverage in PROMs in their entirety [94, 95].

### Limitations of this review

Due to the rarity of LSDs, the studies evaluated did not cover every possible LSD. The LSDs identified in studies within the review may encompass patients susceptible to specific treatments e.g. enzyme replacement therapy or gene therapy. As a result, the framework may provide a narrower overview of the impact of the broad LSD groups (*n*=14) identified, upon HRQoL, as opposed to all possible LSDs receiving all types of treatment. However, the framework may be utilised as a resource to map the impact of HRQoL in other and additional LSDs, where studies become available.

Articles published prior to 2017 were not included in the review and therefore some PROMs which have been used to assess HRQoL in people living with LSDs prior to 2017 may have been missed. However, the time frame restriction was imposed to ensure that the focus of the review was on concepts and domains considered relevant within the contemporary HRQoL literature. Older measures may not include concepts which have more recently been considered relevant and HRQoL domains are likely to continue to receive attention when evidence continues to indicate their relevance. It is therefore unlikely that highly relevant domains of HRQoL have been disregarded as a result of the restriction.

The restriction to full text articles published in English was also imposed and this may have limited the domains identified within the review, thus future work in this area may consider implementing a broader inclusion criteria. These necessary restrictions were imposed in line with best practise guidance [15] due to the rapid scoping review design which was most appropriate given that the aim of the review was to scope the evidence on which HRQoL PROMs have been used in LSDs and to map the results into thematic domains of HRQoL.

Relatedly, since the focus of the review was on the identification of the PROMs utilised and subsequently the HRQoL domains assessed in people with LSDs, quality checks on the individual studies included in the review were not carried out since the approach taken was inclusive of all studies (according to the inclusion criteria) in order to gain a complete record of the PROMs utilised. The quality of individual studies was therefore not relevant to the aims or outcomes of the review.

## Conclusion

A vast range of PROMs have been utilised to assess the broad range of HRQoL outcomes in people with LSDs, including several generic preference based HRQoL instruments and LSD-specific PROMs. Within individual studies, multiple PROMs are frequently used to assess HRQoL. Owing to the range of instruments used to measure HRQoL both across and within the different LSD disease groups, it is challenging to synthesise the studies and to summarise the impact of LSDs on HRQoL. Nevertheless, we have developed an initial conceptual framework of HRQoL for people with LSDs, which includes 37 sub-domains, categorised into: i) Activities; ii) Physical sensations; iii) Autonomy; iv) Cognition; v) Feelings and emotions; vi) Self-identity; and vii) Relationships. This novel framework provides a resource which highlights the HRQoL domains currently measured in the LSD space and acts as information source for researchers and clinicians to identify PROMs for use to measure target aspects of HRQoL in LSDs. The framework may also be used to map the HRQoL domains from existing instruments, identify gaps in coverage according to the priorities of people living with LSDs, and as a platform to more formally assess the validity and reliability of available instruments used in the measurement of HRQoL within LSD populations.

### Supplementary Information

Additional file 1: Contains Appendix A. Search strategy and Appendix B: Additional Tables (including: Table 1 Inclusion and exclusion criteria of PROMs; Table 2 PROMs per LSD group). 

Additional file 2: Contains Appendix C. Papers (all data extracted from the included studies); Appendix D PROMs + Themes (all data extracted from the PROMs); Appendix E Themes by paper (HRQoL themes covered by each study) and Appendix F Excluded PROMs.

## Data Availability

The author confirms that all data generated or analysed during this study are included in this published article or supplementary information.

## Abbreviations

HRQoL: Health Related Quality of Life
LSD: Lysosomal Storage Disorders
PROM: Patient-reported outcome measure
QoL: Quality of life
COSMIN: COnsensus-based Standards for the selection of health status Measurement Instruments
ICF: International Classification of Functioning, Disability and Health framework
DS3: Mainz Severity Score index and disease severity scoring system
ADL: Activities of daily living
