# Supplementary material for: Measuring health related quality of life (HRQoL) in Lysosomal Storage Disorders (LSDs): a rapid scoping review of available tools and domains
Source: Orphanet J Rare Dis. 2024 Jul 4;19:252. doi: 10.1186/s13023-024-03256-0 (PMC11225496; doi:10.1186/s13023-024-03256-0)
Supplement: Supplementary file 1 — Additional file 1: Contains Appendix A. Search strategy and Appendix B: Additional Tables (including: Table 1 Inclusion and exclusion criteria of PROMs; Table 2 PROMs per LSD group). [file 13023_2024_3256_MOESM1_ESM.docx]

**Supplementary materials: A review of the quality of life themes in Lysosomal Storage Disorders (LSDs)**

Appendix A: Search strategy

| **#** | **Searches** |
| --- | --- |
| 1 | exp lysosome storage disease/ |
| 2 | ((lysosom* or "lipid storage") adj3 (disease* or disorder*)).mp. |
| 3 | lipidosis/ or globoid cell leukodystrophy/ or mucopolysaccharidosis/ or mucolipidosis type 2/ or mucolipidosis type 3/ or neuronal ceroid lipofuscinosis/ or infantile neuronal ceroid lipofuscinosis/ or mannosidosis/ or aspartylglycosaminuria/ or cholesterol ester storage disease/ |
| 4 | ((farber* or krabbe* or fabry* or schindler* or sandhoff* or tay-sachs or "tay sachs" or gaucher* or niemann-pick* or "niemann pick" or hurler* or scheie* or hunter* or sanfilippo* or morquio* or maroteaux-lamy* or "maroteaux lamy*" or sly* or i-cell* or "i cell*" or batten* or spielmeyer* or kufs* or wolman* or salla* or pompe* or danon*) adj3 (disease* or syndrome*)).mp. |
| 5 | ((farber* or krabbe* or fabry* or schindler* or sandhoff* or tay-sachs or "tay sachs" or gaucher* or niemann-pick* or "niemann pick" or hurler* or scheie* or hunter* or sanfilippo* or morquio* or maroteaux-lamy* or "maroteaux lamy*" or sly* or i-cell* or "i cell*" or batten* or spielmeyer* or kufs* or wolman* or salla* or pompe* or danon*) adj5 (lysosom* or "lipid storage" or lsd or lsds)).mp. |
| 6 | ((ceramidase or galactocerebrosidase or galactosylceramidase or galc or neuraminidase or beta-galactosidase or "beta galactosidase" or alpha-galactosidase or "alpha galactosidase" or naga or alpha-n-acetylgalactosaminidase or hexosaminidase* or beta-glucosidase or "beta glucosidase" or glucocerebrosidase or sphingomyelin* or "lysosomal acid lipase" or saposin or sulfatase or iduronate 2-sulfatase or "heparan sulfamidase" or arsb or asb or "arylsulfatase b" or beta-glucuronidase or "beta glucuronidase" or hyaluronidase or n-acetylglucosamine 1-phosphotransferase or phosphotransferase or sialidase or cathepsin or mannosidase or alpha-mannosidase or beta-mannosidase or aspartylglucosaminidase or alpha-l-fucosidase or "acid maltase" or "glucosidase acid" or "lysosomal-associated membrane" or lamp*) adj3 deficien*).mp. |
| 7 | ((ganglioside* or lipidos* or oligosaccharide* or cystine) adj3 accumulat*).mp. |
| 8 | (sphingolipidos* or galactosialidosis or gangliosidosis or "activator deficiency" or glucocerebroside or glucosylceramide or sulfatidosis or "metachromatic leukodystrophy" or mld or mucopolysaccharidos* or "mps iv*" or mucolipidos* or sialidosis or "pseudo-hurler polydystrophy" or "neuronal ceroid lipofuscinos*" or ncl or incl or santavuori or hagberg-santavuori or jansky-bielschowsky or lincl or jncl or cln* or ancl or "finnish variant" or "late infantile" or "northern epilepsy" or ctsd or alpha-mannosidosis or "alpha-mannosidosis" or beta-mannosidosis or "beta mannosidosis" or aspartylglucosaminuria or fucosidosis or cystinosis or pycnodysostosis or glycogenosis).mp. |
| 9 | (("mucopolysaccharide storage" or mps or "lysosomal transport" or "sialic acid storage" or issd or "glycogen storage" or gsd or "cholester* ester storage") adj3 (disease* or disorder*)).mp. |
| 10 | or/1-9 |
| 11 | health care survey/ or exp questionnaire/ or (survey* or questionnaire*).mp. |
| 12 | ("health related quality of life" or "health related qol" or "health related ql" or hrqol or hql or "health state utilit*" or hsuv*).tw. |
| 13 | (euroqol or "euro qol" or eq5d or "eq 5d" or sf6d or "sf 6d" or "sf 6 dimension*" or "sf six dimension*" or "shortform 6d" or "shortform six dimension*" or "short form 6d" or "short form 6 dimension*" or "short form six dimension*" or sf12 or "sf 12" or "short form 12" or "shortform 12" or "sf twelve" or sftwelve or "shortform twelve" or "short form twelve" or sf36 or "sf 36" or "short form 36" or "shortform 36" or "sf thirtysix" or "sf thirty six" or "shortform thirtysix" or "shortform thirty six" or "short form thirtysix" or "short form thirty six").tw. |
| 14 | ((item adj3 short form) or (item adj3 shortform) or "medical outcomes survey" or "medical outcomes study" or mos or "psychological general wellbeing index" or "psychological general well being index" or pgwb* or "health utilit*" or hui* or "quality of wellbeing" or "quality of well being" or qwb*).tw. |
| 15 | (rosser or "trade off*" or "standard gamble*" or tto* or qaly* or "quality adjusted life year*" or hye* or "health* year* equivalent*" or disutilit* or disbenefit*).tw. |
| 16 | ((preference* or utilit*) adj2 (elicit* or patient* or population* or measure* or based or cost*)).tw. |
| 17 | quality adjusted life year/ or "Quality of Life"/ or Outcome Assessment/ or "quality of life".tw. |
| 18 | (preference based or utilit* or generic preference).tw. |
| 19 | 17 and 18 |
| 20 | *attitude to health/ or *self care/ |
| 21 | ((patient* adj4 (feeling* or emotion* or view* or symptom* or perception* or attribute*)) or "health related quality of life" or "health related qol" or "health related ql" or hrqol or hql or "patient reported outcome*" or "patient-reported outcome*" or prom or proms or "disease reported outcome*" or "quality of life" or "qol" or "outcome measure*" or "health outcome*").tw. |
| 22 | exp interview/ or exp qualitative research/ or (qualitative* or findings or interview*).mp. |
| 23 | (20 or 21) and 22 |
| 24 | or/11-16,19,23 |
| 25 | 10 and 24 |

**Appendix B: Additional Tables**

**Table 1 Inclusion and exclusion criteria of PROMs**

| **Inclusion** | **Exclusion (n=146)** |
| --- | --- |
| - Accessible questionnaire with a set format with questions in English that the study team could access to assess the items of each PROM - Multi-item PROM measuring HRQoL (i.e. not only behaviour or function) in patients with LSD (i.e. not carer or family members) - Provides a quantitative score - Completed by person with LSD or proxy reported (e.g. family member, carer) and does not require clinical input or assessment | - Not used in last 5 years n=84 - Doesn't report HRQOL (e.g. emotional regulation, behaviour, symptoms, activities, and purely functional measures) n=14 - Insufficient information provided in studies on questionnaire, version or scoring n=14 - Unable to acquire questionnaire / instrument not available e.g. retired, replaced or inaccessible due to language n=14 - Requires clinical assessment or clinician reported / combines clinical data n=11 - Not a multi-item PROM n=6 - Doesn't report pwLSD HRQOL (e.g. family, carer QoL ) n=1 - Doesn't provided a quantitative score n=1 - Adaptive questionnaire without a set format n=1 |

Note: The instruments not used in the last 5 years may not meet other criteria if retained in the review (outlined in further bullet points)

Excluded PROMs are detailed in Appendix F

**Table 2 PROMs per LSD group**

|  | **Alpha-mannosidosis** | **Fabry disease** | **Gaucher disease** | **Hunter syndrome** | **Hurler syndrome & Hurler-Sheie syndrome** | **Mucolipidosis III (ML III)** | **Maroteaux-Lamy syndrome (MPS VI)** | **Morquio syndrome (MPS IVA)** | **Mucopolysaccharidosis (unspecified)** | **Nephropathic cystinosis** | **Niemann-Pick disease** | **Pompe disease** | **Sly syndrome (MPS VII)** | **Wolman disease** |
| --- | --- | --- | --- | --- | --- | --- | --- | --- | --- | --- | --- | --- | --- | --- |
| Achenbach system of empirically based assessment (ASEBA) Child Behavior Checklist - Age 1.5-5 |  |  |  |  | Y |  |  |  |  |  |  |  |  |  |
| Achenbach system of empirically based assessment (ASEBA) Child Behavior Checklist - Age 6-18 |  |  |  |  | Y |  |  |  |  |  |  | Y |  |  |
| Achenbach system of empirically based assessment (ASEBA) Adult self-report |  | Y |  |  |  |  |  |  |  |  |  |  |  |  |
| Activity of daily living survey |  |  |  | Y |  |  |  | Y |  |  |  |  |  |  |
| Beck Depression Inventory |  | Y | Y |  |  |  |  |  |  |  |  |  |  |  |
| Beck Depression Inventory - ii |  |  | Y |  |  |  |  | Y |  |  |  |  |  |  |
| Boston Carpal Tunnel Questionnaire |  |  |  | Y | Y | Y |  |  |  |  |  |  |  |  |
| Brief Pain Inventory Short form |  | Y |  |  |  |  |  | Y |  |  |  |  |  |  |
| Brief Pain Inventory |  | Y |  |  |  |  |  |  |  |  |  |  |  |  |
| Centre for Epidemiological Studies – Depression scale |  | Y |  |  |  |  |  |  |  |  |  |  |  |  |
| Childhood Health Assessment Questionnaire | Y |  |  |  |  |  |  |  |  |  |  |  | Y |  |
| Composite Autonomic Symptom Scale 31 |  | Y |  |  |  |  |  |  |  |  |  |  |  |  |
| Eating Assessment Tool |  |  |  |  |  |  |  |  |  | Y |  |  |  |  |
| Epworth Sleepiness Scale |  | Y | Y |  |  |  |  |  |  |  |  |  |  |  |
| EQ-5D-5L | Y |  |  |  |  |  |  | Y |  |  | Y |  |  |  |
| EQ-5D-3L |  | Y | Y |  |  |  |  | Y | Y | Y |  | Y |  |  |
| EQ-5D-Y | Y | Y | Y |  |  |  |  |  | Y | Y | Y | Y |  |  |
| FabryScan questionnaire |  | Y |  |  |  |  |  |  |  |  |  |  |  |  |
| Fatigue Severity Scale |  |  | Y |  |  |  |  |  |  |  |  | Y |  |  |
| Gaucher Disease type-1-specific Patient Reported Outcome Measure (routine monitoring) |  |  | Y |  |  |  |  |  |  |  |  |  |  |  |
| Gaucher Disease type-1-specific Patient Reported Outcome Measure (clinical trials) |  |  | Y |  |  |  |  |  |  |  |  |  |  |  |
| Geriatric Depression Scale |  |  | Y |  |  |  |  |  |  |  |  |  |  |  |
| Health Assessment Questionnaire |  | Y |  |  |  |  |  | Y |  |  |  |  |  |  |
| Hospital Anxiety and Depression Scale |  | Y | Y | Y | Y |  | Y |  |  |  |  | Y |  |  |
| HUI3 | Y |  |  | Y |  |  |  |  |  |  |  |  |  |  |
| Kiddo-KINDL-r |  | Y |  |  |  |  |  |  |  |  |  |  |  |  |
| Kiddy-KINDL-r |  | Y |  |  |  |  |  |  |  |  |  |  |  |  |
| Kid-KINDL-r |  | Y |  |  |  |  |  |  |  |  |  |  |  |  |
| MD. Anderson Dysphagia Inventory |  |  |  |  |  |  |  |  |  | Y |  |  |  |  |
| Michigan Hand Outcomes Questionnaire |  |  |  | Y |  |  | Y |  |  | Y |  |  |  |  |
| MPS questionnaire |  |  |  |  |  |  |  | Y |  |  |  |  |  |  |
| Neuropathic Pain Symptom Inventory |  |  | Y |  |  |  |  |  |  |  |  |  |  |  |
| Non Motor symptom Questionnaire |  |  | Y |  |  |  |  |  |  |  |  |  |  |  |
| NPC quality-of-life questionnaires for children |  |  |  |  |  |  |  |  |  |  | Y |  |  |  |
| NPC quality-of-life questionnaires for adults |  |  |  |  |  |  |  |  |  |  | Y |  |  |  |
| PainDETECT questionnaire of German Research Network on Neuropathic Pain |  | Y |  |  |  |  |  |  |  |  |  |  |  |  |
| Pediatric Quality of Life Inventory 4.0 - Toddler(age 2-4) -parent report |  |  | Y |  |  |  |  |  |  |  |  |  |  | Y |
| Pediatric Quality of Life Inventory 4.0 - Young children (age 5-7) - self |  | Y | Y |  |  |  |  |  |  |  |  |  |  | Y |
| Pediatric Quality of Life Inventory 4.0 - Young children (age 5-7) - proxy |  | Y | Y |  |  |  |  |  |  |  |  |  |  | Y |
| Pediatric Quality of Life Inventory 4.0 - Child (age 8-12) -self or proxy |  | Y | Y |  | Y |  |  |  |  |  |  |  |  | Y |
| Pediatric Quality of Life Inventory 4.0 - Teens (age 13-18) - self or proxy |  | Y | Y |  | Y |  |  |  |  |  |  |  |  |  |
| Pediatric Quality of Life Inventory 4.0 - Young adults (age 18-25) -self or proxy |  | Y | Y |  |  |  |  |  |  |  |  |  |  |  |
| Pediatric Quality of Life Inventory 4.0 - Adults (age 18+) - self or proxy |  | Y | Y |  |  |  |  |  |  |  |  |  |  |  |
| Pediatric Quality of Life Inventory Multi-dimensional Fatigue Scale - Toddler(age 2-4) -parent report |  |  |  |  |  |  |  |  |  |  |  |  | Y |  |
| Pediatric Quality of Life Inventory Multi-dimensional Fatigue Scale - Young children (age 5-7) - self |  |  |  |  |  |  |  |  |  |  |  |  | Y |  |
| Pediatric Quality of Life Inventory Multi-dimensional Fatigue Scale - Young children (age 5-7) - proxy |  |  |  |  |  |  |  |  |  |  |  |  | Y |  |
| Pediatric Quality of Life Inventory Multi-dimensional Fatigue Scale - Child (age 8-12) -self or proxy |  |  |  |  |  |  |  |  |  |  |  |  | Y |  |
| Pediatric Quality of Life Inventory Multi-dimensional Fatigue Scale - Teens (age 13-18) - self or proxy |  |  |  |  |  |  |  |  |  |  |  |  | Y |  |
| Pediatric Quality of Life Inventory Multi-dimensional Fatigue Scale - Young adults (age 18-25) -self or proxy |  |  |  |  |  |  |  |  |  |  |  |  | Y |  |
| Pediatric Quality of Life Inventory Multi-dimensional Fatigue Scale - Adults (age 18+) - self or proxy |  |  |  |  |  |  |  |  |  |  |  |  | Y |  |
| Pediatric Outcomes Data Collection Instrument - adolescent |  |  |  |  | Y |  |  |  |  |  |  |  |  |  |
| Pediatric Outcomes Data Collection Instrument - child |  |  |  |  | Y |  |  |  |  |  |  |  |  |  |
| Pittsburgh Sleep Quality Index |  | Y | Y |  |  |  |  |  |  |  |  |  |  |  |
| Pompe Disease Impact Scale |  |  |  |  |  |  |  |  |  |  |  | Y |  |  |
| Pompe Disease Symptom Scale |  |  |  |  |  |  |  |  |  |  |  | Y |  |  |
| PROMIS - Dyspnea Short Form 10a |  |  |  |  |  |  |  |  |  |  |  | Y |  |  |
| PROMIS- Fatigue Short Form 8a |  |  |  |  |  |  |  |  |  |  |  | Y |  |  |
| PROMIS - Mobility short form v2.0 |  |  |  | Y | Y | Y |  |  |  |  |  |  |  |  |
| PROMIS - Pain Interference Short Form 8a |  |  |  | Y | Y | Y |  |  |  |  |  | Y |  |  |
| PROMIS - peer relations short form V2.0 |  |  |  | Y | Y | Y |  |  |  |  |  |  |  |  |
| PROMIS - Physical Function Short Form 20a |  |  |  |  |  |  |  |  |  |  |  | Y |  |  |
| Quality of vision |  |  |  |  |  |  |  |  |  | Y |  |  |  |  |
| Rasch-built Pompe-specific Activity Scale |  |  |  |  |  |  |  |  |  |  |  | Y |  |  |
| Revised Child Anxiety and Depression scale |  | Y | Y | Y | Y |  | Y |  |  |  |  | Y |  |  |
| Rotterdam Handicap Scale |  |  |  |  |  |  |  |  |  |  |  | Y |  |  |
| SF-36 - version 1 |  | Y | Y |  |  |  |  | Y |  |  |  | Y |  |  |
| SF-36 - version 2 |  | Y |  |  |  |  |  |  |  |  |  | Y |  |  |
| Spielberger State and Trait Anxiety Inventory |  |  | Y |  |  |  |  |  |  |  |  |  |  |  |
| St. George’s Respiratory Questionnaire |  |  |  |  |  |  |  |  |  |  |  | Y |  |  |
| TNO-AZL Questionnaire for Preschool Children's Health-Related Quality of Life |  |  |  |  |  |  |  | Y |  |  | Y |  |  |  |
| WHOQOL-BREF |  |  |  |  |  |  |  |  |  |  |  | Y |  |  |
|  | 4 | 26 | 23 | 9 | 12 | 4 | 3 | 9 | 2 | 6 | 5 | 18 | 8 | 4 |
